# In Vitro Maturation with Leukemia Inhibitory Factor Prior to the Vitrification of Bovine Oocytes Improves Their Embryo Developmental Potential and Gene Expression in Oocytes and Embryos

**DOI:** 10.3390/ijms21197067

**Published:** 2020-09-25

**Authors:** Meritxell Vendrell-Flotats, Tania García-Martínez, Iris Martínez-Rodero, Manel Lopez-Bejar, Jonathan LaMarre, Marc Yeste, Teresa Mogas

**Affiliations:** 1 Department of Animal Medicine and Surgery, Autonomous University of Barcelona, ES-08193 Cerdanyola del Vallès, Spain; meritxell.vflotats@gmail.com (M.V.-F.); taniagarciamartinez@gmail.com (T.G.-M.); iris.martinez@outlook.com (I.M.-R.); 2 Department of Animal Health and Anatomy, Autonomous University of Barcelona, ES-08193 Cerdanyola del Vallès, Spain; manel.Lopez.Bejar@uab.cat; 3 College of Veterinary Medicine, Western University of Health Sciences, Pomona, CA 91766, USA; 4 Department of Biomedical Sciences, Ontario Veterinary College, University of Guelph, Guelph, ON N1G 2W1, Canada; jlamarre@uoguelph.ca; 5 Department of Biology, Institute of Food and Agricultural Technology, University of Girona, ES-17004 Girona, Spain; marc.yeste@udg.edu

**Keywords:** oocyte, cryopreservation, cow, interleukin, cytokine, maternal effect gene, epigenetics, apoptosis, heat shock protein, maternal–embryonic transition, cell count, apoptosis, inner cell mass

## Abstract

Oocyte cryopreservation has a significant impact on subsequent embryonic development. Herein, we investigated whether supplementing in vitro maturation medium with Leukemia Inhibitory Factor (LIF) prior to vitrification affects embryo development and gene expression at different embryo developmental stages. A panel of genes including maternal effect, epigenetics, apoptosis and heat stress was relatively quantified. The results show reduced cleavage rates after vitrification, regardless of the LIF treatment. Although not statistically different from control-vitrified oocytes, oocyte apoptosis and the blastocyst yield of LIF-vitrified oocytes were similar to their non-vitrified counterparts. Vitrification increased oocyte *ZAR1*, *NPM2* and *DPPA3* gene expression while its expression decreased in LIF-vitrified oocytes to similar or close levels to those of non-vitrified oocytes. With a few gene-specific exceptions, vitrification significantly increased the expression of *DNMT3A*, *HDAC1*, *KAT2A*, *BAX* and *BCL2L1* in oocytes and most stages of embryo development, while comparable expression patterns for these genes were observed between LIF-vitrified and non-vitrified groups. Vitrification increased *HSPA1A* expression in oocytes and *HSP90AA1* in 2-cell embryos. Our data suggest that vitrification triggers stage-specific changes in gene expression throughout embryonic development. However, the inclusion of LIF in the IVM medium prior to vitrification stimulates blastocyst development and several other developmental parameters and induces oocytes and embryos to demonstrate gene expression patterns similar to those derived from non-vitrified oocytes.

## 1. Introduction

Over the past several decades, the cryopreservation of bovine oocytes has become an important part of assisted reproductive technologies. Not only does oocyte cryopreservation permit long-term storage of female genetic material, which is useful for endangered species and livestock with high economic value, but it may also facilitate assisted reproductive procedures and international germplasm exchange [1]. Oocyte vitrification has been successfully used for the cryopreservation of oocytes in humans and many other species (reviewed by [2,3,4]). However, embryonic developmental potential from vitrified bovine oocytes is still relatively low with disparate survival and development rates after warming, probably as a consequence of morphological and cytological damage induced by cryopreservation. The basic mechanisms behind the disturbance of oocyte processes through vitrification are likely to be complex and involve the disruption of cellular structures, such as the cellular skeleton and meiotic spindles; the premature release of cortical granules [5,6] leading to zona hardening and impairment of normal fertilization [7]; compromised mitochondrial integrity [8]; changes in oocyte gene expression [9,10,11], and the initiation of oocyte apoptosis [11]. While these injuries threaten the viability and subsequent embryo development of oocytes, few reports have examined the relationship between gene expression and aberrant early embryonic development or on growth retardation of embryos derived from vitrified bovine oocytes.

During the maternal–embryonic transition (MET), the developmental program shifts from maternal to embryonic control; maternal transcripts are degraded, and the embryonic expression of specific genes is required for differentiation and successful implantation. In bovine embryos, MET occurs during the passage from the 8- to 16-cell stage [12]. Epigenome reprogramming, including DNA methylation changes, histone modification, and genomic imprinting, must occur with high fidelity during the early embryonic development. Similarly, gene expression in early-stage embryos relies extensively upon the post-transcriptional control of maternal factors, which are encoded by maternal effect genes and accumulate during oocyte maturation [13]. Oocyte vitrification can influence epigenetic reprograming [10] and compromise the expression of some maternal transcripts in bovine oocytes and early embryos, which may in turn alter or negatively impact the development of preimplantation embryos [14]. Therefore, understanding the mechanisms by which vitrification influences gene expression in oocytes and embryos represents an important step towards understanding the overall impact that vitrification/warming has on embryo developmental potential.

The success of vitrification relies on a delicate balance between the concentration, toxicity and permeability of cryoprotectants (CPAs), sample volume, cooling rate and donor species. Technical approaches designed to improve oocyte vitrification have focused on CPA exposure regimes, cooling/warming rates and distinct storage vessels. Another approach entails specific measures or protocols to improve oocyte quality and cryotolerance during in vitro maturation (reviewed by [3]). In the ovary, follicular fluid provides an optimized environment for maturation to occur, thus affecting the oocyte potential for fertilization and subsequent embryonic development. Previous studies have shown that a variety of growth factors and cytokines can be found in the follicular fluid. Among the members of the family of the interleukin-6 (IL-6) cytokines, leukemia inhibitory factor (LIF) has been shown to have relevant reproductive functions [15]. LIF signals by binding to a heterodimeric membrane receptor complex formed by a LIF-specific receptor (LIFR) and a signal transducing surface protein receptor known as the interleukin-6 receptor subunit beta (IL6ST, also known as GP130). This mechanism is shared by other cytokines of the IL-6 family including Cardiotrophin-1 (CT-1) and IL-6 [16]. The receptor complex mediates downstream LIF signaling via multiple pathways, including JAK/STAT (Janus Kinase/Signal Transducer and Activator of Transcription), MAPK (Mitogen-Activated Protein Kinases) and PI3K/AKT (phosphatidylinositol 3-kinase/AKT serine/threonin kinase) [17]. LIF appears to play key roles during follicular growth and improves in vitro cumulus expansion and oocyte competence, embryo development [18,19,20,21,22], and cellular cryotolerance to vitrification (Kocyigit and Cevik 2015) in different species.

Here, we postulate that oocyte vitrification, as a significant stressor on oocyte viability, is likely to have a significant impact on post-transcriptional regulation of gene expression, epigenetic reprogramming and apoptosis during early embryonic development, which ultimately results in lower developmental potential. Accordingly, the objective of this study was to determine whether the addition of LIF during in vitro maturation could modulate the effects of oocyte vitrification on: (a) oocyte apoptosis and embryo developmental competence after vitrification/warming; (b) the total number of blastomeres together with its allocation to inner cell mass (ICM) or trophectoderm (TE) cells and the apoptosis rate of derived blastocysts; and (c) the relative transcript abundance of maternal-effect genes (MEG) (*ZAR1*, zygote arrest 1; *NPM2*, nucleoplasmin 2; *DPPA3*, developmental pluripotency associated 3), and genes related to epigenetics (*DNMT3A*, DNA Methyltransferase 3 Alpha; *KAT2A*, Histone Acetyltransferase; *HDAC1*, Histone Deacetylase 1), apoptosis (*BAX*, *BCL2L1*) and heat stress (*HSPA1A*, Heat Shock Protein Family A (Hsp70); *HSP90AA1*, Heat Shock Protein 90 Alpha Family A (Hsp90)) in bovine oocytes, early embryos and blastocysts.

## 2. Results

### 2.1. Effects of LIF Supplementation during IVM on Embryo Development, Total Cell Number, ICM and TE Cell Distribution and Apoptosis Rate of Blastocysts Derived from Vitrified/Warmed Oocytes

Table 1 compares the effects of LIF addition during the in vitro maturation of bovine oocytes prior to vitrification/warming on embryo development. Vitrification significantly decreased embryo development at 48 h post-insemination (cleavage rate) when compared to the control non-vitrified group, regardless of the LIF treatment. Oocytes vitrified after LIF treatment produced similar percentages of Day 7 and Day 8 blastocysts as fresh non-vitrified oocytes in vitro matured with or without LIF. Although percentages of Day 7 and Day 8 blastocysts resulting from vitrified oocytes IVM (in vitro maturation) without LIF supplementation were significantly lower than those obtained from fresh control oocytes, they did not differ from both fresh and vitrified LIF treated oocytes. IVM in the presence of LIF significantly increased the hatching rates of Day 8 blastocysts when compared to the vitrified groups, but no significant differences were observed between non vitrified groups.

**Figure 1:**
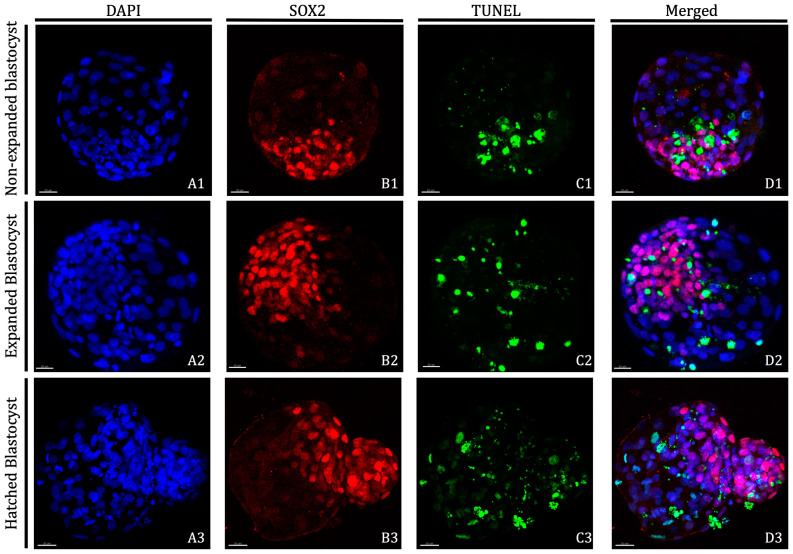
Representative images of D8 non-expanded, expanded and hatched blastocysts derived from vitrified/warmed oocytes in vitro matured in the presence of 25 ng/mL of LIF. Nuclei counterstained with DAPI are displayed in blue (**A1**–**A3**), Inner Cell Mass is displayed in red (**B1**–**B3**) and TUNEL-positive cells are displayed in green (**C1**–**C3**). Overlayed images are presented in **D1**–**D3**. Non-expanded blastocysts: (**A1**,**B1**,**C1**,**D1**); Expanded blastocysts: (**A2**,**B2**,**C2**,**D2**); and Hatched blastocysts: (**A3**,**B3**,**C3**,**D3**). Scale bar: 30 μm. DAPI (406-diamidino-2-phenylindole), TUNEL (Terminal deoxynucleotidyl transferase dUTP nick end labelling).

Total cell number (TCN), number of ICM cells, number of TE cells and apoptotic rate (AR) of Day 8 blastocysts derived from oocytes in vitro matured in the presence of 25 ng/mL of LIF prior to vitrification/warming are shown in Table 2 and images obtained after differential cell staining of blastocysts are displayed in Figure 1. Blastocysts derived from oocytes vitrified/warmed after IVM with LIF supplementation showed similar TCN and number of TE cells than the non-vitrified groups, regardless of the blastocyst stage. However, both values were significantly lower for blastocysts derived from vitrified oocytes in vitro matured without LIF supplementation, although no differences were observed between both vitrified groups. While in vitro maturation with LIF supplementation produced blastocysts with significantly higher numbers of ICM cells when compared to blastocysts derived from oocytes matured without LIF, blastocysts derived from both vitrified groups showed similar ICM cell numbers as blastocysts derived from both non-vitrified groups. Furthermore, a significant increase in TCN, ICM cell number and TE cell number was observed as the blastocyst stage progressed from non-expanded to hatched, with the greatest number evident at hatching/hatched stages. No differences in apoptosis rates were observed in blastocysts derived from fresh non-vitrified oocytes, regardless of the LIF treatment. Although apoptosis rate was significantly higher in blastocysts derived from vitrified groups compared to non-vitrified groups, expanded and hatched blastocysts derived from vitrified oocytes previously matured in presence of LIF produced a significantly lower ratio of apoptosis than their vitrified counterparts.

### 2.2. Effects of LIF Supplementation during IVM on the Percentages of TUNEL-Positive Oocytes after Vitrification/Warming

**Figure 2:**
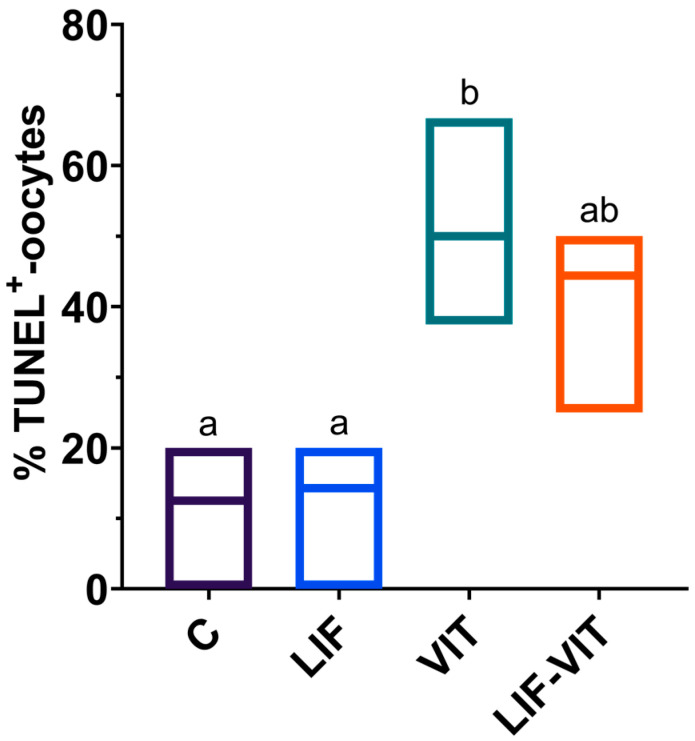
Effect of LIF supplementation during in vitro matured (IVM) previously to vitrification/warming on the percentage of TUNEL-positive oocytes. ^a,b^ Values with different letters differ significantly (*p* < 0.05). Values are presented as box plots (median and Q1/Q3) from three separate experiments. Control: In vitro-matured oocytes (*n* = 25); LIF: In vitro-matured oocytes in medium supplemented with LIF (*n* = 25); VIT: In vitro-matured oocytes vitrified/warmed by the cryotop method (*n* = 22); LIF-VIT: In vitro-matured oocytes in medium supplemented with LIF and vitrified/warmed by the cryotop method (*n* = 25).

**Figure 3:**
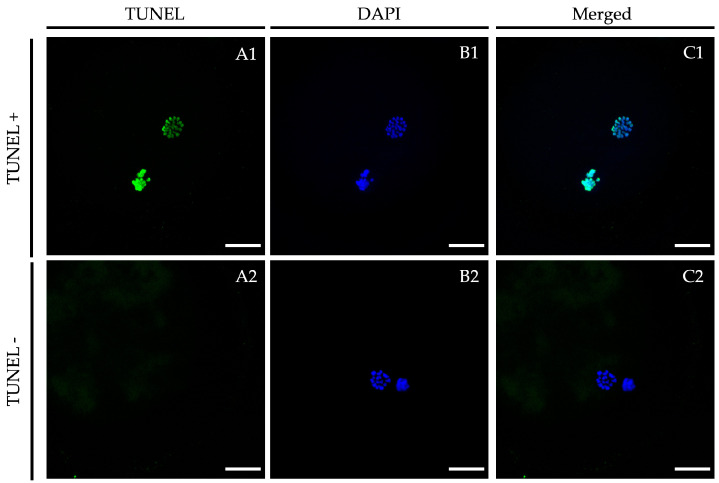
Representative TUNEL assay images vitrified/warmed oocytes after in vitro maturation in the presence of LIF. Oocytes were stained by TUNEL to detect apoptosis ((**A1**,**A2**); green signal) and nuclei were counterstained with DAPI ((**B1**,**B2**); blue signal). (**A1**,**B1**): TUNEL-positive oocyte; (**A2**,**B2**): TUNEL-negative oocyte. An overlay is given in (**C1**,**C2**). Scale bar: 20 μm. DAPI (406-diamidino-2-phenylindole), TUNEL (Terminal deoxynucleotidyl transferase dUTP nick end labeling).

Significantly higher percentage of vitrified/warmed oocytes demonstrated DNA fragmentation when compared with their untreated counterparts. Although not significantly different from the vitrified group, percentages of TUNEL-positive oocytes in the LIF-vitrified group were similar to those of fresh non-vitrified treatments (Figure 2 and Figure 3).

### 2.3. Effects of LIF Supplementation during IVM on Gene Expression of Metaphase II Oocytes and Early Embryos Derived from Vitrified/Warmed Oocytes

**Figure 4:**
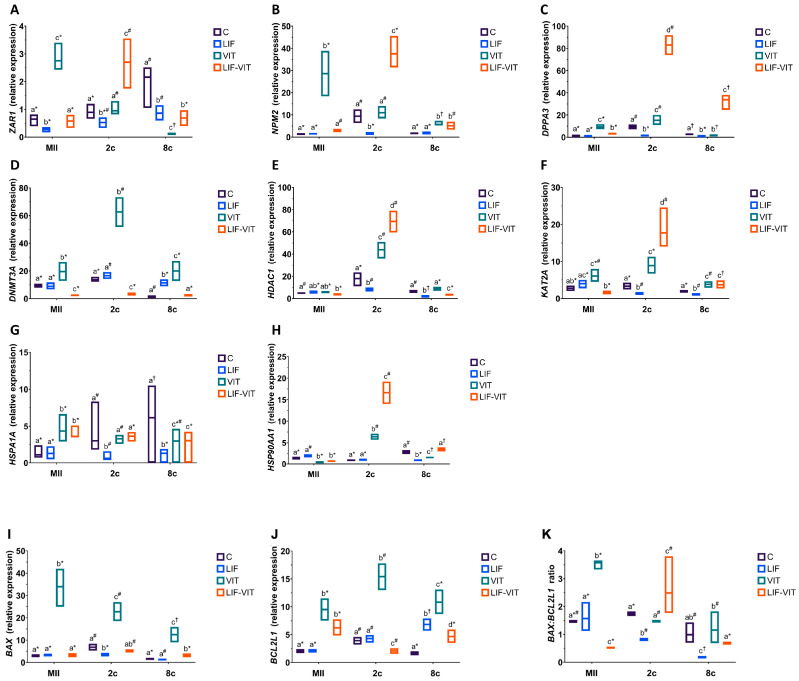
(**A**) *ZAR1*, (**B**) *NPM2*, (**C**) *DPPA3* (**D**) *DNMT3A*, (**E**) *HDAC1,* (**F**) *KAT2A,* (**G**) *HSPA1A*, (**H**) *HSP90AA1,* (**I**) *BAX*, (**J**) *BCL2L1* profiles and (**K**) *BAX:BCL2L1* ratio in metaphase II (MII) oocytes, 2- and 8-cell embryos from control fresh (C), LIF supplemented (LIF), control vitrified (VIT) and LIF supplemented-vitrified (LIF-VIT) in vitro-matured bovine oocytes. The expression levels of these genes are shown as box plots (median and Q1/Q3) from 3 independent experiments. Different letters indicate statistically significant differences between treatments (*p* < 0.05), and different symbols indicate differences between developmental stages inside each specific treatment. *ZAR1*, zygote arrest 1; *NPM2*, nucleoplasmin 2; *DPPA3*, developmental pluripotency associated 3; *DNMT3A*, DNA methyltransferase 3 alpha; *KAT2A*, Lysine acetyltransferase 2A; *HDAC1*, Histone deacetylase 1; *HSPA1A*, Heat shock 70 kDa protein; *HSP90AA1*, Heat shock protein HSP 90-alpha; *BAX*, BCL2-associated X apoptosis regulator; *BCL2L1*, BCL2-like 1; MII, metaphase II; 2c, 2-cell embryos; 8 c, 8-cell embryos.

When mRNA expression was analyzed in in vitro-matured oocytes, vitrification significantly increased the expression of MEG *ZAR1, NPM2* and *DPPA3*, whereas oocytes vitrified after in vitro maturation in medium supplemented with LIF showed similar (*ZAR1* and *NPM2*) or slightly higher (*DPPA3*) gene expression levels as control fresh oocytes (Figure 4A–C). In contrast, the expression of *ZAR1, NPM2* and *DPPA3* significantly increased in 2-cell embryos derived from LIF-vitrified oocytes when compared to the other treatment groups. For 8-cell embryos, vitrification decreased the expression of *ZAR1* and *DPPA3* genes and increased that of *NPM2* when compared to 8-cell embryos derived from control metaphase II (MII) oocytes. In vitro maturation with LIF prior to vitrification significantly increased mRNA expression of *NPM2* and *DPPA3* genes in 8-cell embryos. In addition, whereas levels of *ZAR1* were similar to those observed in 8-cell embryos derived from LIF-IVM oocytes, they were significantly lower than those observed in control fresh oocytes.

With regard to transcript abundance for genes implicated in epigenetic processes, vitrification significantly increased mRNA levels for *DNMT3A* in oocytes and early embryos when compared to the other treatment groups (Figure 4D). MII oocytes and 2-cell embryos derived from LIF-vitrified oocytes showed significantly lower levels of *DNMT3A* abundance compared to the other treatment groups, whereas the expression levels for this gene in 8-cell derived embryos from LIF-vitrified oocytes were similar to the control group. While 2-cell embryos derived from vitrified and LIF-vitrified oocytes significantly increased *HDAC1* transcript levels, significantly lower HDCA2 abundance was observed in both MII oocytes and 8-cell embryos derived from LIF-vitrified oocytes (Figure 4E). Vitrification caused a significant increase in *KAT2A* expression in MII oocytes and early-stage embryos while the abundance of mRNA *KAT2A* transcripts was significantly lower in MII oocytes and significantly higher in 2-cell and 8-cell embryos derived from LIF-vitrified oocytes when compared to fresh control groups (Figure 4F).

Levels of mRNA for *HSPA1A* were significantly greater in MII oocytes and lower in 8-cell derived embryos from both vitrified groups when compared to the fresh control group (Figure 4G). In contrast, the relative abundance of *HSP90AA1* was significantly lower in MII oocytes and significantly greater in 2-cell embryos derived from both vitrified groups when compared to both control groups. While 8-cell embryos derived from LIF and control vitrified groups exhibited significantly lower expressions of *HSP90AA1*, no differences in the relative transcript abundance of this gene were observed between 8-cell embryos derived from fresh control and LIF-vitrified oocytes (Figure 4H)

With regard to the expression of apoptosis-related genes, vitrification significantly increased *BAX* transcript levels in MII and early embryos when compared to the other treatments, whereas LIF treatment prior to vitrification maintained *BAX* gene expression at levels similar to those of fresh control groups (Figure 4I). Similarly, levels of *BCL2L1* gene expression were significantly higher in MII oocytes and early embryos derived from both vitrified groups. However, the abundance of *BCL2L1* transcripts was significantly increased in MII oocytes and 8-cell embryos derived from LIF-vitrified oocytes compared to the control fresh groups, whereas its expression was decreased in 2-cell embryos (Figure 4J). Together, these changes resulted in a *BAX:BCL2L1* ratio that was significantly higher for vitrified MII oocytes and significantly lower for those oocytes vitrified after LIF treatment, when compared to both fresh control groups (Figure 4K). Furthermore, whereas 2-cell and 8-cell embryos derived from non-vitrified control and vitrified groups had similar *BAX:BCL2L1* ratios, this ratio was significantly lower in 2-cell and 8-cell embryos derived from LIF-treated oocytes. LIF-vitrified oocytes produced 2-cell embryos with significantly higher *BAX:BCL2L1* ratios while 8-cell derived embryos showed a similar ratio when compared to the non-vitrified control group.

### 2.4. Effects of LIF Supplementation during IVM on Gene Expression of In Vitro-Produced Blastocysts Derived from Vitrified/Warmed Oocytes

**Figure 5:**
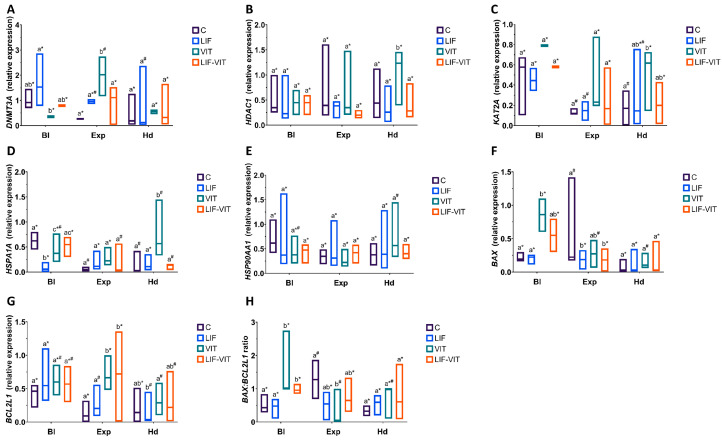
(**A**) *DNMT3A*, (**B**) *HDAC1*, (**C**) *KAT2A*, (**D**) *HSPA1A*, (**E**) *HSP90AA1*, (**F**) *BAX*, (**G**) *BCL2L1* profiles and (**H**) *BAX:BCL2L1* ratio in blastocysts derived from control fresh (C), LIF supplemented (LIF), control vitrified (VIT) and LIF supplemented-vitrified (LIF-VIT) In vitro-matured bovine oocytes. The expression levels of these genes are shown as box plots (median and Q1/Q3) from 3 independent experiments. Different letters indicate statistically significant differences between treatments (*p* < 0.05), and different symbols indicate differences between developmental stages inside each specific treatment. *DNMT3A,* DNA methyltransferase 3 alpha; *KAT2A,* Lysine acetyltransferase 2A; *HDAC1* Histone deacetylase 1; *HSPA1A*, Heat shock 70 kDa protein; *HSP90AA1*, Heat shock protein HSP 90-alpha; *BAX*, BCL2-associated X apoptosis regulator; *BCL2L1,* BCL2-like 1; Bl, Non-expanded blastocyst; Exp, Expanded blastocyst; Hd Hatching/Hatched blastocyst.

Changes in specific gene expression observed in in vitro-produced blastocysts derived from vitrified/warmed oocytes after LIF supplementation during IVM are shown in Figure 5. When mRNA levels for specific transcripts were analyzed in D8 blastocysts, vitrification/warming significantly increased *HSPA1A* and *BAX* expression in blastocysts, *DNMT3A* and *BCL2L1* expression in expanded blastocysts and *HDAC1* and *KAT2A* expression in hatched blastocysts when compared to blastocysts derived from fresh control oocytes. By contrast, vitrified/warmed oocytes produced blastocysts with a significantly lower *DNMT3A* mRNA levels (blastocyst) and a lower *BAX*:*BCL2L1* ratio (expanded blastocysts). However, blastocysts derived from oocytes vitrified/warmed after LIF treatment showed similar levels of gene expression to blastocysts derived from control fresh oocytes, regardless of the blastocyst stage, with the exception of *BAX* and *BCL2L1* genes that were suppressed (*BAX*) or increased (*BCL2L1*) in expanded blastocysts derived from vitrified/warmed oocytes IVM with LIF. However, no differences in the *BAX*:*BCL2L1* ratio were observed between blastocysts derived from control fresh and LIF-vitrified oocytes, regardless of the developmental stage.

## 3. Discussion

Oocyte quality, a key factor determining blastocyst yield, can be directly affected by in vitro maturation [23]. This is particularly significant in the context of cryopreservation, where a major obstacle to the use of vitrified oocytes is the impact of freezing on oocyte quality. As a result, many approaches have been attempted to modify the in vitro maturation protocols of oocytes destined for vitrification in order to increase cryotolerance. These include modifications of plasma membrane permeability and cytoplasmic lipid content, the use of antioxidants and the exposure of oocytes to sublethal stressors prior to vitrification (reviewed in [3]). However, vitrification of bovine oocytes remains an ineffective technique due to their low ability to undergo proper embryonic development after warming and subsequent fertilization.

The main objective of this study was to evaluate whether supplementing IVM medium with LIF (25 ng/mL) increases the resilience of oocytes to withstand vitrification and warming. To this end, we determined blastocyst development and quality in terms blastocyst TCN, distribution of ICM and TE cells and incidence of oocyte and blastocyst apoptosis together with the expression levels of several key genes after in vitro fertilization and culture. While some studies [22] have indicated significantly higher cleavage rates and blastocyst yields, in our study the total cleavage and blastocyst rates after LIF treatment were not different from controls, as observed by Wasielak et al. [24] in pigs. Similar to the results reported by Mo et al. [22], a significantly higher number of ICM cells and a clear trend towards higher hatching ability was consistently observed in blastocysts derived from oocytes matured in vitro with LIF. Although vitrification produced significantly lower cleavage and blastocyst rates, vitrification of bovine oocytes after in vitro maturation in the presence of LIF resulted in similar blastocyst rates to fresh non-vitrified oocytes, although no significant differences were actually observed between the vitrified groups. Moreover, blastocysts derived from the LIF-vitrified group exhibit similar TCN and number of ICM and TE cells as the non-vitrified groups, regardless of the blastocyst stage. It is well documented that the optimal allocation of the blastocyst cells into ICM and TE is crucial for embryonic survival and post-implantation development, as it is has been recognized that a defined minimal number of ICM cells is required for a pregnancy, and excessive allocation of cells to the TE may lead to early pregnancy failures [25,26].

The present study also examined the effects of in vitro maturation with LIF prior to vitrification/warming on the mRNA level of MEG (*ZAR1*, *NPM2* and *DPPA3*) in bovine MII oocytes and early embryo stages. During oocyte maturation, the process of transcription, which is highly active during oocyte growth, becomes markedly decreased. The fully grown oocyte itself is essentially transcriptionally silent, although recent data do suggest that some small degree of transcriptional activity may still occur in this period [27]. Therefore, oocyte developmental competence relies on both the constitution of an adequate pool of RNA in immature oocytes and highly orchestrated regulation of the stability and processing of these mRNAs during maturation before transcription resumes during embryonic genome activation (EGA). In bovine species, two waves of EGA are recognized: a minor increase in transcription in 2- to 4-cell stage embryos [28,29] and a major wave at the 8- to 16-cell stage. The earlier wave activates gene transcription for RNA processing, translation and transport factors. During the second EGA wave, genes involved in chromatin structure, transcription, RNA processing, protein biosynthesis, signal transduction, cell adhesion and maintenance of pluripotency become transcribed. In the present study, RT-qPCR (quantitative reverse transcription PCR) analysis revealed that the expression of *ZAR1*, *NPM2* and *DPPA3* genes is constant throughout in vitro development, regardless of the addition of LIF to the IVM medium, as already described in pigs [24]. A significantly greater abundance of *ZAR1*, *NPM2* and *DPPA3* transcripts was observed in vitrified IVM oocytes, whereas similar or slightly higher transcript levels for these genes was found in oocytes vitrified after LIF-IVM when compared to fresh control oocytes. Oxidative stress during oocyte maturation increases the expression of *DPPA3* in in vitro-matured mouse oocytes and the abundance of DPPA3 protein in the early embryo [30]. One might therefore attribute the higher abundance of *DPPA3* transcripts observed in 2-cell and 8-cell embryos derived from LIF-vitrified oocytes to an enhanced response to the oxidative stress induced by vitrification/warming. However, the increased mRNA abundance of *ZAR1* and *NPM2* in early-stage embryos derived from oocytes vitrified after LIF-treatment could also be related to elevated transcription directed at protecting stressed cells, although no studies confirming this hypothesis are currently found in the literature.

During oocyte and embryo development, epigenetic modifications regulate specific levels of gene expression via chromatin remodeling, histone modifications and DNA methylation [31]. DNA methylation occurs at cytosine residues and is mediated by DNA methyltransferases (DNMT: *DNMT3A*, *DNMT3B* and *DNMT3L*) [32]. Studies in bovine have shown that DNMT3 is required for establishing maternal DNA methylation imprints during oocyte growth and early embryonic development [33,34,35]. Aberrations in germline reprogramming of maternal and/or paternal imprinted loci can be associated with reduced embryonic survival. While we found that the expression of *DNMT3A* was increased in vitrified IVM matured oocytes, 2-cell and 8-cell embryos and expanded blastocysts when compared to non-vitrified treatments, this increase was not observed when the IVM medium was supplemented with LIF. This may underlie observed aberrations in germline reprogramming of maternal and/or paternal imprinted loci in embryos from oocytes subjected to freezing and may be associated with reduced embryonic survival, since significantly lower cleavage and blastocyst yields were observed after vitrification. Moreover, *DNMT3A* was significantly decreased in blastocysts derived from vitrified oocytes, which is consistent with previous bovine studies [10]. Together, these data suggest that oocyte vitrification is accompanied by alterations in DNA methylation in bovine oocytes and embryos, which may contribute to impaired embryonic development and lower embryo quality observed after vitrification. Our data also suggest that LIF has the potential to partially ameliorate or alter these effects. Importantly, differences in DNMT3A-transcript levels observed between treatment groups were no longer evident when blastocysts reached the hatched stage, which suggests that only embryos which are correctly “programmed” are competent to complete preimplantation development (Yuan et al. 2003).

In addition to DNA methylation, the steady-state level of histone acetylation is another crucial mechanism that affects transcriptional regulation. Two enzyme families, histone acetyltransferases (HATs/KAT) and histone deacetylases (HDACs) regulate the acetylation of lysine residues of core histone proteins and thereby influence chromatin remodeling, DNA accessibility and gene expression [36]. In this study, independent of LIF treatment, vitrification increased the expression of *KAT2A* in MII oocytes and led to higher transcript abundance of *HDAC1* and *KAT2A* in 2-cell stage embryos followed by a decrease at 8-cell stage embryos. Modified histone acetylation levels have been described after vitrification in mouse [37,38], cow [10] and pig [39] oocytes. In addition, increased expression of *HDAC1* and *KAT2A* at the 2-cell stage may result from a minor round of transcription after fertilization but before the onset of the major embryonic genome activation. Notably, anomalously elevated histone acetylation has been associated with poor oocyte and embryo quality [40,41]. Therefore, the increased *HDAC1* and *KAT2A* levels in 2-cell stage embryos may be another marker of impairment due to oocyte vitrification. Furthermore, while greater levels of *HDAC1* and *KAT2A* expression were also observed in hatching blastocysts derived from vitrified oocytes, those levels in hatched blastocysts derived from LIF-vitrified oocytes were similar to those in fresh control groups. Differences in some histone modifications are known to differ between the ICM and TE, leading to implantation failure [42]. Reduced and comparable-to-normal levels of expression at the hatched blastocyst stage may indicate either the release of a repressive stage or an unidentified selection process operating during culture, such that only appropriately programmed embryos reach advanced stages.

Apoptosis is a widely recognized and highly regulated form of cellular death resulting from different classes of cellular injury. The process is tightly controlled by a genetic program. *BAX* is an apoptosis-stimulating protein [43], whereas BCL-2 is antiapoptotic, with expression preventing cellular apoptosis [44]. The ratio between these two proteins helps determine whether a cell survives or undergoes apoptosis. We found that vitrification increased *BAX* and *BCL2L1* expression in MII oocytes. In embryos that developed from vitrified oocytes, levels of *BAX* and *BCL2L1* remained high. Surprisingly, the ratio still favored a higher *BAX* level, suggesting a persistent tendency towards apoptosis to the 8-cell stage. Similar to our results, vitrification of IVM bovine oocytes has been previously reported to increase the *BAX*:*BCL2L1* ratio, mainly due to significantly increased *BAX* mRNA levels [11,45]. Moreover, changes in the expression of apoptotic genes in vitrified oocytes may interfere with subsequent embryo development, as most apparently normal vitrified-warmed oocytes fail to develop during the first days of culture and undergo degeneration [3,46]. Our results also show that IVM with LIF significantly decreased the expression of *BAX* and increased *BCL2L1* expression, suggesting that LIF may protect the oocyte from intrinsic apoptosis, presumably by limiting *BAX* and likely via the activation of STAT/JAK [47] and/or PI3K/AKT pathways [48]. Although no differences in cleavage rate and blastocyst yield were observed between vitrified and LIF-vitrified oocytes, development to the blastocyst stage for LIF-vitrified oocytes was similar to that observed for fresh control oocytes. The results also show that oocytes vitrified/warmed after in vitro maturation with LIF showed lower percentages of TUNEL-positive oocytes compared to vitrified oocytes, but similar levels to those of fresh non-vitrified oocytes, which may explain why LIF-vitrified oocytes have a higher developmental competence when compared to their vitrified counterparts [49]. While the *BAX*:*BCL2L1* ratio was significantly lower for expanded blastocysts derived from vitrified oocytes, no differences between treatments were observed at the hatched stage. It is worth noting that, when apoptosis was evaluated by TUNEL labelling to detect apoptosis in preimplantation bovine embryos, we found that blastocysts derived from oocytes vitrified/warmed after IVM with LIF showed lower incidences of apoptotic cells than their vitrified counterparts. Although apoptosis is a normal process in preimplantation embryos to eliminate compromised cells, a high incidence of apoptosis indicates an abnormal morphology of the embryo [50]. Therefore, the lower apoptosis rate together with a lower, but not significant, *BAX*:*BCL2L1* ratio in hatched blastocytes [49] may provide an explanation for why only those embryos derived from oocytes vitrified/warmed after IVM with LIF were able to hatch.

Heat shock proteins (HSPs) have a critical role in the recovery of cells from stressful or cytotoxic environmental stimuli, protecting them from subsequent insults. Expression of HSPs is considered to be one indicator of oxidative and thermal stress as they are induced to enhance resistance against apoptosis, facilitating protein refolding and assembly, and regulating the cellular redox state. Increased expression of HSPs in cells experiencing stress provides them with a functional repair pathway and increases survival (reviewed in [51]). Here, we focused on the expression of *HSPA1A* and *HSP90AA1*, which encode two abundantly expressed and highly conserved HSPs: HSP70 and HSP90. Relative abundance of *HSPA1A* transcripts was significantly higher in vitrified-warmed oocytes and blastocysts, independent of LIF supplementation. Higher *HSPA1A* expression has already been observed after vitrification of human immature oocytes [52]. However, vitrification of canine oocytes [53] or prepubertal bovine oocytes does not modify *HSPA1A*-expression. With respect to *HSP90AA1* expression, significantly lower levels were observed in vitrified oocytes, in a similar fashion to that reported in vitrified-warmed Yak oocytes [54]. However, vitrified groups exhibited a significant increase in *HSP90AA1* transcript levels at 2-cell stage, which was further increased after LIF treatment. Cardiotrophin 1 (CD-1) and LIF have each been shown to increase the relative abundance of HSP70 and HSP90 proteins in neonatal cardiomyocytes, thus protecting them against the exposure to severe thermal or ischemic stress [55,56]. Another cytokine, interleukin-6 (IL-6), increases HSP70 and HSP90 protein levels in liver cells [57]. Based on the significant overlap in their receptor binding and cellular signaling pathways [16], one possible explanation for the higher expression of *HSP90AA1* could be that LIF is capable of protecting cells from stressful stimuli via the activation of the STAT/JAK pathway, as has been demonstrated in liver cells, where IL-6 family members and STAT-3 modulate the expression of HSPs genes [56]. No differences in *HSP90AA1* expression were observed in later stages of embryo development, regardless of the LIF treatment or vitrification.

In conclusion, the current study has identified differences in the expression profiles of several developmentally relevant genes during the brief time period encompassing blastocyst formation and hatching in embryos derived from vitrified oocytes. Increased or decreased expression of genes related to embryo genome activation, epigenetics, apoptosis or heat stress in vitrified oocytes and embryos derived from them could result from aberrant utilization or stability of specific maternal transcripts during oocyte maturation or after fertilization. This may, in turn, affect oocyte quality and gene expression in the in vitro-produced embryos that develop. However, the addition of LIF to maturation media prior to vitrification had a positive influence on blastocyst production, and the expression of factors implicated in maternal effect and epigenetic reprogramming. At the same time, lower expression of apoptotic markers was observed under these conditions. Our study also suggests that further research directed at the mechanisms through which LIF affects oocyte maturation and embryo development and interrogating whether LIF influences specific aspects of oocyte maturation is clearly warranted.

## 4. Materials and Methods

### 4.1. Chemicals and Suppliers

All chemicals and reagents were purchased from Sigma Chemical Co. (St. Louis, MO, USA) unless otherwise stated.

### 4.2. Oocyte Collection and In Vitro Maturation

The in vitro protocols followed for maturation have been described previously by Rizos et al. [58]. Bovine ovaries were transported from a local slaughterhouse in phosphate-buffered saline (PBS) at 35–37 °C. Cumulus-oocyte complexes (COCs) were obtained by aspirating 3–10 mm follicles. Only COCs with three or more layers of cumulus cells and a homogeneous cytoplasm were selected for in vitro maturation. After three washes in modified Dulbecco’s PBS (PBS supplemented with 0.036 mg/mL pyruvate, 0.05 mg/mL gentamicin and 0.5 mg/mL bovine serum albumin (BSA), groups of up to 20 COCs were placed in 100 µL drops of maturation medium and cultured for 24 h at 38.5 °C in a 5% CO_2_ humidified air atmosphere. The maturation medium consisted of TCM-199 supplemented with 10% (*v/v*) fetal bovine serum (FBS), 10 ng/mL epidermal growth factor and 50 µg/mL gentamicin.

### 4.3. Oocyte Vitrification and Warming

The vitrification–warming procedure was essentially that described by Morató et al. [6].

#### 4.3.1. Vitrification Protocol

The holding medium (HM) used to formulate vitrification/warming solutions consisted of Hepes-TCM199 supplemented with 20% (*v/v*) FBS. Partially denuded oocytes were transferred to equilibration solution (ES: 7.5% (*v/v*) ethylene glycol (EG) and 7.5% (*v/v*) dimethyl sulfoxide (DMSO) in HM) for 9 min. Subsequently, oocytes were transferred into vitrification solution (VS: 15% (*v/v*) DMSO, 15% (*v/v*) EG and 0.5 M sucrose in HM). After incubating for 30–40 s, groups of six oocytes were loaded onto a Cryotop (Dibimed-Biomedical Supply, S.L., Valencia, Spain); almost all the solution was removed to leave only a thin layer covering the oocytes, and the device was subsequently plunged into liquid nitrogen. The entire process from exposure in vs. to plunging was completed with less than 90 s.

#### 4.3.2. Warming Protocol

Vitrified oocytes from both experimental groups were warmed after one hour in liquid nitrogen by directly immersing the Cryotop device into 1 M sucrose dissolved in HM for 1 min. Oocytes were then transferred to HM containing 0.5 M sucrose for 3 min and then incubated in HM alone for 5 min. Oocytes were then transferred back into maturation drops at 38.5 °C in humidified air with 5% CO_2_ to allow them to recover for 2 additional hours.

### 4.4. In Vitro Fertilization and Embryo Culture

In vitro-matured oocytes were in vitro fertilized and cultured as previously described by Arcarons et al. [59]. Briefly, high motility and good morphology spermatozoa were obtained by centrifuging frozen/thawed sperm from Asturian bulls (ASEAVA, Llanera, Asturias, Spain) at 300× *g* and room temperature for 10 min on a discontinuous gradient (1 mL of 40% and 1 mL of 80% BoviPure; Nidacon Laboratories AB, Göteborg, Sweden) according to the manufacturer’s instructions. Viable spermatozoa were collected from the bottom, washed with 3 mL of BoviWash (Nidacon International, Göteborg, Sweden) and pelleted by centrifugation at 300× *g* for 5 min. Spermatozoa were counted in a Neubauer chamber and diluted in an appropriate volume of fertilization medium (Tyrode’s medium supplemented with 25 mM bicarbonate, 22 mM Na-lactate, 1 mM Na-pyruvate, 6 mg/mL fatty acid-free BSA and 1 mg/mL heparin-sodium salt) to a final concentration of 1 × 10^6^ spermatozoa/mL. One-hundred-microliter droplets of diluted sperm were prepared under mineral oil and 20 oocytes/droplet were co-incubated at 38.5 °C, 5% CO_2_ and high humidity.

After 18–20 h, presumptive zygotes were stripped of remaining cumulus cells by pipetting and cultured in groups of 20 in 20-µL drops of IVC (in vitro culture) medium, consisting of Synthetic Oviduct Fluid (Caisson Labs, Smithfield, UT, USA) supplemented with 0.96 μg/mL BSA, 88.6 μg/mL Na-pyruvate, 2% non-essential amino acids, 1% essential amino acids, 0.5% gentamicin and 2% FBS under mineral oil. Presumptive zygotes were incubated at 38 °C in a humidified 5% CO_2_ and 5% O_2_ atmosphere for 8 days. Embryo development was recorded on Day 2 (cleavage) and Days 7 and 8 (blastocysts) post-insemination (pi). Day 8 embryos were classified in terms of the degree of blastocoel expansion into three groups according to the IETS (International Embryo Technology Society) standards: (a) early blastocysts (stage code 5) and blastocysts (stage code 6); (b) expanded blastocysts (stage code 7) and (c) hatching (stage code 8) or hatched blastocysts (stage code 9).

### 4.5. Differential Staining of Blastocysts

Day 8 bovine blastocysts underwent double staining combined with TUNEL analysis as previously described by Ascari et al. [60] with some modifications; all steps were done at 38.5 °C unless otherwise stated. Blastocysts were fixed in 2% (*v/v*) paraformaldehyde in PBS for 15 min at room temperature (RT). After fixation, embryos were washed at least three times in PBS and permeabilised in 0.01% Triton X-100 in PBS supplemented with 5% Normal Donkey Serum (PBS-NDS) for 1 h at RT. The embryos were washed in PBS (×3) and incubated at 4 °C overnight with mouse anti-SOX2 primary antibody (1:100; Invitrogen, CA, USA) in a humidified chamber. Afterward, the embryos were washed in PBS (×3) for 20 min and permeabilized again with 0.005% Triton X-100 in PBS-NDS for 20 min. The embryos were then incubated with the secondary antibody goat anti-mouse IgG AlexaFluor568 (1:500; ThermoFisher, Waltham, MA, USA) diluted for 1 h in a humidified chamber. Afterwards, the embryos were transferred to PBS-NDS-0.005% Triton X-100 for 20 min, washed in PBS (×3) and incubated in the TUNEL reaction mixture dilution following manufacturer’s instructions (in situ Cell Death Detection Kit, Fluorescein) for 1 h in the dark. Positive and negative control samples were included in each assay. Blastocysts exposed to DNase I (1 U/mL) for 15 min at RT served as positive controls and blastocysts not exposed to the terminal TdT enzyme served as negative controls. Embryos were then washed thoroughly in 0.005% Triton X-100 in PBS-NDS for 5 min, mounted on poly-L-lysine treated coverslips fitted with a self-adhesive reinforcement ring in a 3-µL drop of Vectashield containing 125 ng mL^−1^ 4′,6-diamidino-2-phenylindole (DAPI) (Vectorlabs, Burlingame, CA, USA), and flattened with a slide. The preparation was sealed with nail varnish and stored at 4 °C protected from light until observation within the following up to 3 days. Confocal images in serial sections separated by 0.5 µm were captured with a confocal laser scanning microscope (Leica TCS SP5, Leica Microsystems CMS GmbH, Mannheim, Germany) to examine an ICM cell’s nucleus (SOX2-Alexa Fluor 568; excitation 561 nm), cell nucleus (DAPI; excitation 405 nm) and DNA fragmentation (fluorescein isothiocyanate-conjugated TUNEL label; excitation 488 nm). TCN, ICM cell number, and apoptotic cells were analyzed using the Imaris 9.2 software (Oxford Instruments, UK). Individual nuclei were counted and assessed as intact (TUNEL(−); blue/red stain) or fragmented (TUNEL(+), green stain) DNA, TE cells (SOX2(−); blue stain) or ICM cells (SOX2(+); red stain) (Figure 1). The AR was calculated as the ratio of TUNEL(+) cells/total number of cells.

### 4.6. TUNEL Assay in Oocytes

After 24 h of maturation, oocytes from fresh and vitrified/warmed treatment groups were completely denuded of cumulus cells by gentle pipetting in hyaluronidase (1000 UI in PBS) to remove the cumulus cells and submitted to the TUNEL assay as described in Sprícigo et al. [9] with some modifications. Briefly, oocytes were fixed in 4% (*w/v*) paraformaldehyde in PBS for 1 h at RT. After fixation, they were washed (×3) in PBS containing polyvinylpyrrolidone (0.3% PVP in PBS; PBS-PVP) and permeabilized in 0.5% (*v/v*) Triton X-100 containing 0.1% (*w/v*) sodium citrate for 1 h at RT. The oocytes were then washed (×3) in PBS-PVP and incubated in the TUNEL reaction cocktail following the manufacturer’s instructions (in situ Cell Death Detection Kit, Fluorescein) at 38.5 °C for 1 h in the dark. Oocytes exposed to DNase I (50 mL of RQ1 RNase-free Dnase (50 U/mL)) for 1 h at RT served as positive controls, and oocytes not exposed to the terminal TdT enzyme served as negative controls. Oocytes were mounted on poly L-lysine-treated coverslips fitted with a self-adhesive reinforcement ring in a 3-μL drop of Vectashield containing 125 ng/mL 4,6-diamidino-2-phenylindole hydrochloride (DAPI) (Vectorlabs, Burlingame, CA, USA) and flattened with a coverslip. Preparations were sealed with nail varnish and stored at 4 °C protected from light until observation within the following 2 days. An epifluorescence microscope (Axioscop 40FL; Carl Zeiss, Göttingen, Germany) was used to examine TUNEL-positive nuclei (fluorescein isothiocyanate-conjugated TUNEL label; excitation BP 450–490; FT 510; LP 515–565), while nuclei were localized using the DAPI filter (DAPI; excitation BP 365/12; FT 395; LP 397). Nuclei were scored as having either intact (TUNEL [−]; blue stain) or fragmented (TUNEL [+]; green stain) DNA (Figure 3). The percentage of TUNEL-positive oocytes was calculated as the ratio between TUNEL(+) oocytes and the total number of analyzed oocytes in each group.

### 4.7. RNA Extraction, Reverse Transcription and Quantitative Real-Time PCR Analysis

RNA extraction, reverse transcription and quantitative real-time PCR analysis were performed as described [59]. Samples for gene expression analysis were collected in groups of 20 oocytes or 5 embryos, plunged into liquid nitrogen and stored at −80 °C. Poly-(A)-RNA was extracted using the Dynabeads mRNA Direct Extraction Kit (Invitrogen, Oslo, Norway), following the manufacturer’s instructions with minor modifications. For poly-(A)-RNA extraction, pooled samples were lysed in 50 μL Lysis Buffer for 5 min. In order for hybridization to occur, the fluid lysate was incubated with 10 μL pre-washed beads for 5 min. Poly-(A)-RNA-bead complexes were washed twice in 50 μL Washing Buffer A and two further times in 50 μL of Washing Buffer B. Next, samples were eluted in 16 μL Elution Buffer (Tris HCl) and heated to 70 °C for 5 min.

Reverse transcription (RT) reaction was carried out with 4 μL qScript cDNAsupermix (Quanta Biosciences; Gaithersburg, MD, USA), using oligo-dT primers, random primers, dNTPs and qScript reverse transcriptase. After RT, the cDNA was diluted with 25 μL of Tris HCl. The RT reaction was performed for 5 min at 25 °C, followed by 1 h at 42 °C to allow the RT of mRNA, and 10 min at 70 °C to denature the reverse transcriptase enzyme.

Quantification of relative abundance of mRNA transcripts was performed by the qPCR method using a 7500 Real-Time PCR System (Applied Biosystems, Foster City, California, USA) and 10 μL Fast SYBR Green Master Mix (Applied Biosystems, Foster City, CA, USA). A 2-μL cDNA template was used for each reaction. To verify the identity of the amplified PCR product, melting curve analysis and gel electrophoresis (in a 2% agarose gel containing 0.6 μg/mL ethidium bromide) were performed. Three technical replicates, each coming from the same biological sample and individual gene, were evaluated. Furthermore, negative controls for the template and for the reverse transcription were also included and amplified by qPCR to ensure that no cross-contamination occurred.

Ten candidate genes (*ZAR1*; *NPM2*; *DPPA3*; *DNMT3A*; *KAT2A*; *HDAC1*; *BAX*; *BCL2L1*; *HSPA1A*; *HSP90AA1*) for GV (germinal vesicle), MII, 2-cell and 8-cell embryos and seven genes (*DNMT3A*; *KAT2A*; *HDAC1*; *BAX*; *BCL2L1*; *HSPA1A*; *HSP90AA1*) for different blastocyst stages were evaluated using RT-qPCR analysis; *GAPDH* and *H2AFZ* (endogenous control genes) were used to normalize quantification. Fold differences in relative transcript abundance were calculated for target genes assuming an amplification efficiency of 100% and using the formula 2^−(ΔΔ*C*T)^ [61]. Calculation of ΔΔ*C*T involved the subtraction of the Δ*C*T value for the untreated (GV in experiment 1 and early blastocysts for experiment 2) from all the other Δ*C*T sample values. Primer sequences (Life Technologies, Madrid, Spain), amplicon sizes and GenBank accession numbers for each gene are provided in Table 3. Non-template controls were not amplified or returned a *C*T value 10 points higher than the average *C*T value for all genes.

### 4.8. Experimental Design

#### 4.8.1. Effects of LIF Supplementation during IVM on Embryo Development, TCN, ICM and TE Cell Distribution and Apoptosis Rate of Blastocysts Derived from Vitrified/Warmed Oocytes

**Figure 6:**
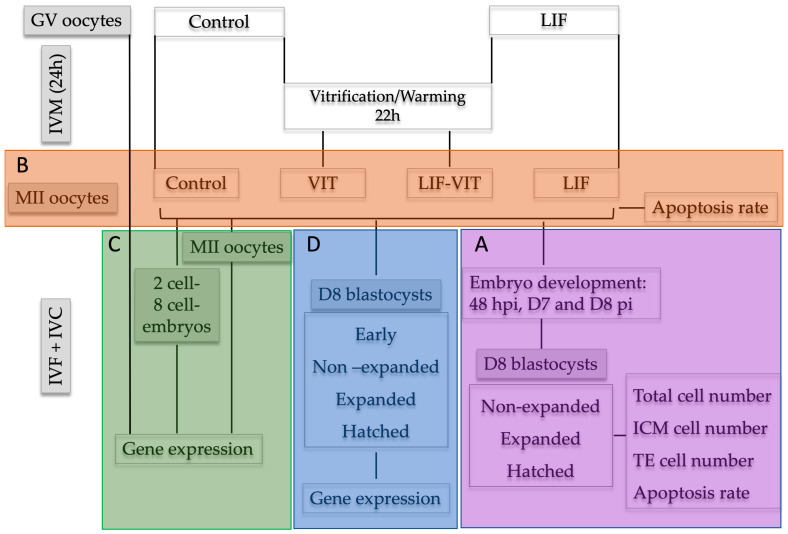
Experimental design. Upon oocyte collection, immature cumulus-oocyte complexes (COCs) were randomly divided into two experimental groups: (**A**) control group: oocytes were in vitro-matured as previously described; and (**B**) LIF group: oocytes were in vitro matured using the same IVM medium supplemented with human recombinant LIF (25 ng/mL). After 22 h, half of the in vitro-matured oocytes from Control and LIF groups were vitrified/warmed and allowed to recover for 2 h (VIT and LIF-VIT groups, respectively). Experimental group (**A**): Oocytes were in vitro fertilized and cultured. Cleavage rates were assessed at 48 hpi and blastocyst rates on Day 7 and Day 8 pi. D8 embryos were classified in non-expanded, expanded and hatched blastocysts and immunostained to assess TCN, ICM cell number, TE cell number and AR. Experimental group (**B**): At 24 h of IVM, oocytes were denuded and immunostained to assess the percentage of apoptosis by TUNEL assay. Experimental group (**C**): GV oocytes collected a 0 h, and MII oocytes, 2-cell and 8-cell embryos collected from each group at 24 h of IVM, 31–33 hpi and 52–54 hpi, respectively, were stored at −80 °C for RT-qPCR analysis. Experimental group (**D**): Oocytes were in vitro fertilized and cultured up to day 8 pi. Day 8 embryos were classified as early, non-expanded, expanded and hatched blastocysts and stored at 80 °C for RT-qPCR analysis.

Upon oocyte collection, immature COCs were randomly divided into two experimental groups: (a) control (*n* = 615), oocytes were in vitro-matured as previously described; and (b) LIF (*n* = 630)**,** oocytes were in vitro-matured using the same IVM medium supplemented with human recombinant LIF (25 ng/mL) [22]. After 22 h, half of the in vitro-matured oocytes from the control and LIF groups were vitrified/warmed and allowed to recover for 2 h (VIT (*n* = 302) and LIF-VIT (*n* = 318) groups, respectively). Oocytes were then in vitro fertilized and cultured. Cleavage rates were assessed at 48 hpi and blastocyst rates on day 7 and day 8 pi. At day 8, embryos were classified into three groups, non-expanded (stage code 5–6), expanded (stage code 7), and hatched blastocysts (stage code 8–9) and fixed and immunostained to assess TCN, ICM cell number, TE cell number and AR (Figure 6A).

#### 4.8.2. Effects of LIF Supplementation during IVM on the Percentages of TUNEL-Positive Oocytes after Vitrification/Warming

Upon oocyte collection, immature COCs were randomly divided into two experimental groups: (a) control, oocytes were in vitro matured as previously described; and (b) LIF, oocytes were in vitro matured using the same IVM medium supplemented with human recombinant LIF (25 ng/mL). After 22 h, half of the in vitro-matured oocytes from the control and LIF groups were vitrified/warmed and allowed to recover for 2 h (VIT and LIF-VIT groups, respectively). At 24 h of IVM, oocytes from each group were denuded from cumulus cells by gentle pipetting and a total of 97 oocytes demonstrating extrusion of the first polar body were fixed and immunostained to assess the percentage of apoptosis by TUNEL assay in 3 replicates (7–10 oocytes/replicate) (Figure 6B).

#### 4.8.3. Effects of LIF Supplementation during IVM on Gene Expression of Metaphase II Oocytes and Early Embryos Derived from Vitrified/Warmed Oocytes

Upon oocyte collection, immature COCs were randomly allocated into three experimental groups: (a) GV, immature oocytes at germinal vesicle stage; (b) control, oocytes were in vitro matured as previously described; and (c) LIF, oocytes were in vitro matured using the same IVM medium supplemented with human recombinant LIF (25 ng/mL). After 22 h, half of the in vitro-matured oocytes from the control and LIF groups were vitrified/warmed and allowed to recover for 2 additional hours (VIT and LIF-VIT groups, respectively). Upon collection, GV oocytes were denuded mechanically from cumulus cells. At 24 h of IVM, a pool of oocytes from each group was denuded from cumulus cells by gentle pipetting and 20 oocytes demonstrating extrusion of the first polar body and thereby presumed to be in MII were collected from each group. The remaining oocytes were in vitro fertilized and cultured. Two-cell and 8-cell embryos were harvested from the culture medium at 31–33 hpi and 52–54 hpi, respectively. All oocyte groups as well as 2-cell and 8-cell embryos were snap frozen in liquid nitrogen and stored at −80 °C until RNA isolation and RT-qPCR analysis were performed. Three independent experiments were conducted (Figure 6C).

#### 4.8.4. Effects of LIF Supplementation during IVM on Gene Expression of In Vitro-Produced Blastocysts Derived from Vitrified/Warmed Oocytes

Upon oocyte collection, immature COCs were randomly divided into two experimental groups: (a) control, oocytes were in vitro matured as previously described; and (b) LIF, oocytes were in vitro matured using the same IVM medium supplemented with human recombinant LIF (25 ng/mL). After 22 h, half of the in vitro-matured oocytes from the control and LIF groups were vitrified/warmed and allowed to recover for 2 h (VIT and LIF-VIT groups, respectively). Oocytes were then in vitro fertilized and cultured up to Day 8 pi. For gene analysis, Day 8 embryos were classified as early (stage code 5), blastocyst (stage code 6), expanded (stage code 7), and hatched (stage code 9), snap frozen in liquid nitrogen and stored at 80 °C until RNA isolation and RT-qPCR analysis were performed. Three independent experiments were conducted (Figure 6D).

### 4.9. Statistical Analyses

Statistical tests were performed using the statistical package IBM SPSS Version 25.0 for Windows (IBM Corp.; Armonk, NY, USA). Data were first checked for normal distribution (Shapiro–Wilk test) and homogeneity of variances (Levene test). When required, data were linearly transformed through √x or arcsin √x, prior to running two-way analysis of variance ANOVA (factors: stage and treatment) followed by post-hoc Sidak test for pair-wise comparisons. The variables examined were: cleavage rates, blastocyst yields, percentage of TUNEL-positive cells, TCN, ICM and TE cell number, AR and relative transcript abundance. When linear transformations did not establish normal distributions and/or homoscedasticity in the data, Scheirer–Ray–Hare and Mann–Whitney tests were used as non-parametric alternatives. Data are expressed as percentage ± SD, mean ± SD, and median and Q1/Q3. Significance was set at *p* ≤ 0.05.

## Figures and Tables

**Table 1:** Developmental competence of bovine oocytes in vitro matured in a medium supplemented with leukemia inhibitory factor (LIF) prior to vitrification/warming.

					D8 Blastocyst			
	*n*	Cleavage Rate	D7 Blastocyst	D8 Blastocyst	*n*_D8_	Non-Expanded	Expanded	Hatched
Control	275	90.42 ± 3.15 ^a^	27.16 ± 7.33 ^a^	37.42 ± 9.39 ^a^	105	13.01 ± 6.01 ^a,b^	35.84 ± 9.42 ^a^	51.15 ± 13.16 ^a,b^
LIF	293	77.85 ± 8.72 ^a,b^	26.53 ± 8.89 ^a,b^	31.13 ± 5.80 ^a,b^	90	6.66 ± 7.86 ^a^	32.59 ± 8.00 ^a^	60.75 ± 10.98 ^a^
VIT	200	70.95 ± 14.3 ^b^	13.8 ± 12.07 ^b^	17.48 ± 8.80 ^b^	33	30.00 ± 26.47 ^b^	31.67 ± 7.64 ^a^	38.33 ± 18.93 ^b^
LIF-VIT	223	72.81 ± 13.67 ^b^	16.70 ± 7.21 ^a,b^	25.85 ± 8.90 ^a,b^	47	16.05 ± 11.47 ^a,b^	44.00 ± 15.16 ^a^	39.96 ± 14.36 ^b^

*n* = number of oocytes that were fertilized at 24 h of IVM; D7, D8: day 7 or day 8 post-insemination. ^a,b^ Different superscript letters within a column are significantly different values (*p* < 0.05). Values given as percentage ± SD. Control: In vitro-matured oocytes. LIF: In vitro-matured oocytes in medium supplemented with LIF. VIT: In vitro-matured oocytes vitrified/warmed by the cryotop method; LIF-VIT: In vitro-matured oocytes in medium supplemented with LIF and vitrified/warmed by the cryotop method.

**Table 2:** TCN, number of cells in the ICM, TE and AR of Day 8 blastocysts derived from oocytes in vitro matured in media supplemented with LIF prior to vitrification/warming.

				Day 8 Blastocysts											
	*n*			TCN ± SD			ICM Cell Number ± SD			TE Cell Number ± SD			AR ± SD		
	Bl	Exp	Hd	Bl	Exp	Hd	Bl	Exp	Hd	Bl	Exp	Hd	Bl	Exp	Hd
Control (*n* = 30)	9	10	11	92.4 ± 8.0 ^a,d^	150.1 ± 9.9 ^a,e^	189.3 ± 12.8 ^a,f^	9.1 ± 3.1 ^a,d^	17.2 ± 2.9 ^a,e^	27.4 ± 5.4 ^a,f^	83.3 ± 3.6 ^a,d^	132.9 ± 6.6 ^a,e^	161.9 ± 14.9 ^a,f^	9.7 ± 0.5 ^a,d^	4.5 ± 0.3 ^a,e^	4.2 ± 0.7 ^a,e^
LIF (*n* = 30)	8	11	11	96.7 ± 5.7 ^a,d^	147.8 ± 8.5 ^a,e^	197.4 ± 10.6 ^a,f^	13.5 ± 1.9 ^b,d^	21.6 ± 3.5 ^b,e^	34.9 ± 4.2 ^b,f^	83.2 ± 4.2 ^a,d^	127.2 ± 8.1 ^a,e^	162.5 ± 11.6 ^a,f^	8.9 ± 1.2 ^a,d^	3.8 ± 0.2 ^a,e^	4.1 ± 0.9 ^a,e^
VIT (*n* = 19)	5	8	6	72.2 ± 8.1 ^b,d^	129.5 ± 17.0 ^b,e^	177.1 ± 18.9 ^b,f^	10.1 ± 3.6 ^a,b,d^	20.5 ± 6.2 ^a,b,e^	22.6 ± 9.2 ^a,b,f^	62.4 ± 8.5 ^b,d^	106.9 ± 15.1 ^b,e^	154.4 ± 18.0 ^b,f^	16.2 ± 2.6 ^b,d^	12.1 ± 2.3 ^b,e^	13.0 ± 1.2 ^b,e^
LIF-VIT (*n* = 24)	7	8	9	91.3 ± 9.0 ^a,b,d^	139.5 ± 15.2 ^a,b,e^	182.2 ± 17.5 ^a,b,f^	12.7 ± 4.2 ^a,b,d^	22.3 ± 5.0 ^a,b,e^	30.3 ± 7.1 ^a,b,f^	78.6 ± 7.1 ^a,b,d^	117.2 ± 12.3 ^a,b,e^	151.9 ± 15.6 ^a,b,f^	17.4 ± 2.3 ^c,d^	10.6 ± 3.6 ^c,e^	8.9 ± 1.9 ^c,e^

^a,b,c^ Values within columns with different superscripts differ significantly (*p* < 0.05); ^d,e,f^ Values within rows for each valuable with different superscripts differ significantly (*p* < 0.05). Data shown as mean ± SD. Control: In vitro-matured oocytes. LIF: In vitro-matured oocytes in medium supplemented with LIF. VIT: In vitro-matured oocytes vitrified/warmed by the cryotop method; LIF-VIT: In vitro-matured oocytes in medium supplemented with LIF and vitrified/warmed by the cryotop method. TCN: Total cell number; ICM: Inner Cell Mass; TE: Trophectoderm; AR: Apoptotic rate. Bl, Non-expanded blastocyst; Exp, Expanded blastocyst; Hd Hatching/Hatched blastocyst.

**Table 3:** Primers used for reverse transcription–quantitative polymerase chain reaction.

Symbol	NCBI Gene Name	Gene Bank Accession Number	Primer Set Sequences (5′-3′)	Amplicon Size (bp)
*BAX*	BCL2-associated X, apoptosis regulator	NM_173894.1	F: GAGAGGTCTTTTTCCGAGTGGC	237
			R: TGTCCCAAAGTAGGAGAGGAG	
*BCL2L1*	BCL2-like 1	BC147863.1	F: CCACTTAGGACCCACTTCTGAC	188
			R: GGGTGCTTCCTACAGCTACAGT	
*DNMT3A*	DNA methyltransferase 3 alpha	NM_001206502.1	F: CCTCAGCTCCCCCTACTTATTC	199
			R: AGCTGTGAGCTTACTCCTGAGC	
*DPPA3*	Developmental pluripotency associated 3	NM_001111108.2	F: TGGCTACTCTTCATCCCCTACA	230
			R: TCTAGGGTCCAGGTTGGGTT	
*GADPH*	Glyceraldehyde-3-phosphate dehydrogenase	NM_001034034.2	F: AGTCCACTGGGGTCTTCACTAC	243
			R: CAGTGGTCATAAGTCCCTCCAC	
*HDAC1*	Histone deacetylase 1	NM_001037444.2	F: CTGAGGAGATGACCAAGTACC	167
			R: CCACCAGTAGACAGCTGACAGA	
*HSPA1A*	Heat shock protein family A (Hsp70) member 1A	NM_203322.3	F: GCAGGTGTGTAACCCCATCA	181
			R: CAGGGCAAGACCAAAGTCCA	
*HSP90AA1*	Heat shock protein 90 alpha family class A member 1	NM_001012670.2	F: GTGGAGACTTTCGCCTTCCA	223
			R: TGGTGAGGGTTCGATCTTGC	
*H2AFZ*	H2A histone family, member Z	NM_174809.2	F: GCGTATTACCCCTCGTCACTTG	227
			R: GTCCACTGGAATCACCAACACTG	
*KAT2A*	Lysine acetyltransferase 2A	XM_015468132.1	F: AGGATGTGGCTACCTACAAGG	190
			R: GCACCAGCTTGTCCTTCTCTAC	
*NPM2*	Nucleophosmin/Nucleoplasmin 2	NM_001168706.1	F: GGACCTGTGTTCCTCTGTGG	153
			R: CTTCACTTGTTTGACGGGCG	
*ZAR1*	Zygote arrest 1	NM_001076203.1	F: GGGAGATGCAAAGGCAAACG	216
			R: CCAAACAACAGCCTTCCACG	
